# Scanning Electron Microscopic Evaluation of the Sealer-Dentine Interface of Three Sealers

**DOI:** 10.22037/iej.2017.08

**Published:** 2017

**Authors:** Fatemeh Mohammadian, Farzad Farahanimastary, Fatemeh Dibaji, Mohammad Javad Kharazifard

**Affiliations:** a *Department of Endodontics, Dental School, International Campus of Tehran University of Medical Sciences, Tehran, Iran; *; b * Dental School, International Campus of Tehran University of Medical Sciences, Tehran, Iran; *; c * Dental Research Center, Dentistry Research Institute, Tehran University of Medical Sciences, Tehran, Iran*

**Keywords:** Endodontic Sealer, Interface, Gap, Root Sealer, Scanning Electron Microscopy

## Abstract

**Introduction:**

This study aimed to evaluate the dentine-sealer interface in three different sealers using scanning electron microscopy (SEM).

**Methods and Materials:**

Thirty extracted human single-rooted teeth were prepared using ProTaper rotary files and were randomly divided into three groups (*n*=10) including BC Sealer, AH-Plus and Dorifill. The root canals were filled with cold lateral condensation technique and stored for 7 days in 100% humidity at 37^°^C. Cross sections were prepared from the coronal, middle, and apical sections of the roots. Then SEM images were taken and the width of gaps was measured by software. Sectional images were evaluated by two endodontists. Data were analyzed using two- and one-way ANOVA and Kruskal-Wallis tests.

**Results:**

The mean gap width was significantly lower in coronal area in BC Sealer group compared to Dorifill (*P*=0.043) and likewise in AH-Plus group compared to Dorifill (*P*=0.018). There was no significant difference between BC Sealer and AH-Plus group in this area (*P*=0.923). No significant difference was detected in apical and middle zones among three sealers (*P*=0.367 and 0.643, respectively). Dentine-sealer interface showed no significant difference in three sealers in the apical area (*P*=0.051), but dentine-BC Sealer interface was better than AH-Plus in middle and coronal areas, and both outperformed Dorifill (*P*=0.001).

**Conclusion:**

BC Sealer and AH-Plus had less gaps than Dorifill in coronal area. In addition, BC Sealer had better dentine interface in middle and coronal area compared to AH-Plus, and both performed better than Dorifill. Reverse relationship was observed between the mean gap width and dentine-sealer interface quality.

## Introduction

The aim of root canal therapy is treatment of periapical lesions or prevention of their development [1, 2]. Poorly filled areas of root canal system can be a source of bacteria with growth ability, which obtain nutrition from periapical area [3]. Thus dense and three dimensional root canal filling must be provided with gutta-percha cones and root canal sealers [4]. Sealers are mandatory for long term seal of the root canal obturation [5]. Sealers are capable of filling the voids between gutta-percha cones and the gap between gutta-percha and dentinal canal walls [6, 7]. Sealers may be made based on different materials such as zinc oxide eugenol (ZOE), calcium hydroxide, glass ionomer, epoxy resin [8]. 

In different kinds of sealers, ZOE-based sealers are usually chosen for reasonable affordability, easy access and bacteriostatic property [9] and AH-plus is commonly selected because of its good adhesion to the dentine, penetration in tubules and adaptation to the peritubular dentine [10].

Recently, Bioceramic sealers based on a calcium phosphate silicate composition has been introduced as a new group of sealers [11]. Endosequence BC Sealer (Brasseler USA, Savannah, GA, USA) is a premixed bioceramic endodontic sealer [12]. Its main inorganic components include tricalcium silicate, dicalcium silicate, calcium phosphates, colloidal silica and calcium hydroxide [13]. This sealer is biocompatible, non-toxic and stable in biological environments. Because of the highly alkaline pH (≥12) during the setting process, Endosequence BC Sealer has bactericidal properties [14]. In addition, This bioceramic sealer is hydrophil, uses the inside moisture of dentinal tubules for setting and does not tolerate shrinkage during setting time and hardening [15]. On the other hand, this sealer has the ability to produce hydroxyapatite crystals during setting time and finally creates a chemical bond between the filling material and root dentine [16].

Few studies have been conducted about the quality of dentine interface of this sealer compared to other sealers [17, 18]. The aim of this study was to determine dentine-sealer interface of BC Sealer and compare it with an epoxy resin-based sealer (AH-Plus) and a ZOE-based sealer (Dorifill) using scanning electron microscopy (SEM).

## Materials and Methods

***Preparation of specimens***

Thirty recently extracted human single-rooted premolars without caries, apical resorption, root surface resorption and cracks were selected. In order to preserve humidity of dentinal tubules, the teeth were stored in saline solution. Soft tissue residues and calculi were removed using a scaler (insert perio, Sonicflex 2000, KaVo, Biberach, Germany). For disinfection, all samples were stored in refrigerator for seven days in a solution of 0.5% chloramine-T at a temperature of 4^°^C. Then, the crowns were cut using a 014 fissure diamond bur (Teeskavan Co., Tehran, Iran) under copious water irrigation to yield roots sections with 13-mm lengths. 

Working length was determined using a #10 K-file (Mani Co, Utsunomiya, Tochigi, Japan). The file was inserted into the canal until the tip was visible at the apex; one millimeter was subtracted from this length to determine the working length. The root canals were prepared using ProTaper rotary files (Dentsply Maillefer, Ballaigues, Switzerland) using the single length technique up to F3 (30/0.09). Between each file, canals were rinsed with 3 mL of 2.5% sodium hypochlorite (NaOCl). After completion of instrumentation, 1 mL of 17% ethylenediaminetetraacetic acid (EDTA) (Prime Dental products, India) was used for 1 min to remove the smear layer followed by 3 mL of 5.25% NaOCl. Final rinse was done with 5 mL of distilled water. The canals were then dried with paper points.

***Obturation of the root canals***

The roots were randomly divided into three groups to be filled with either of the three sealers including BC Sealer (Brasseler USA, Savannah, GA, USA), AH-Plus sealer (Dentsply**,** DeTrey, Konstanz, Germany) and Dorifill sealer (Dorident Company, Vienna, Austria) and gutta-percha (Aryadent, Tehran, Iran) using lateral condensation filling technique. In BC Sealer group the premixed sealer was placed into the canal with the syringe and some was also dispensed on the mixing paper pad for covering the master cone. In AH-Plus group sealer was mixed in equal volumes (1:1) of pastes A and B (Epoxide and Amine pastes) on a mixing pad to a homogenous consistency. In the third group Dorifill sealer was also prepared according to the manufacturer’s instruction. A standard #30 master cone was selected and confirmed using a radiography. Then the cone was coated with sealer and introduced into the apical area. In all groups obturation was continued by lateral compaction technique.

To allow complete setting of sealer, the teeth were stored in 100% humidity at 37^°^C for 7 days. Then the roots were transversely sectioned by 200 µm-thick diamond blade (CNC, Fanavaran Pars, Mashhad, Iran). Sample thickness was 2 mm and distance of the first cut to coronal surface and last cut to apex tip was 1.5 mm. 

***Sample preparation for SEM evaluation***

Cut sections were dehydrated for observation by SEM. Following mounting on aluminum stubs; they were coated by a thin layer of gold in a coater system. Then, each cross section was divided into four equal parts under microscope and images were prepared under 300× magnification.

Width of the existing gaps were measured by Image Tool software (UTHSCSA software, University of Health Sciences San Antonio, Texas). Dentine-sealer interface was qualitatively recorded from images by two blinded and calibrated endodontists. Ranking was done as follows: presence of 0 to 2 small gaps with <2µm width (good), 3 to 4 small gaps (reasonable) and more than 4 small or large-sized gaps (poor). 

Findings were statistically analyzed using the two-and one-way ANOVA in order to compare gap width in experiment groups in different areas, and the Tukey’s test was used for pairwise comparison of the groups. Also, the Kruskal-Wallis test was used for evaluation of dentine-sealer interface at different areas and Donn’s test was applied for pairwise comparison of the groups.

## Results

Minimum, maximum and mean of gap values in experimental groups are given in Table 1. Considering the significance of statistical interaction effect of sealer type and area factors on the gap width (*P*=0.043), no difference was observed in apical area among three types of sealers in gap width (*P*=0.367). There was no difference among three types of sealers in terms of gap width (*P*=0.643) in middle area. However, a significant difference was detected in coronal area. Dorifill had significantly higher gap widths compared to AH-Plus (*P*=0.018) and BC sealer (*P*=0.043) in coronal, but there was no significant difference between AH-Plus and BC Sealer in this area (*P*=0.923).

No significant difference was between three types of sealers in terms of dentine-sealer interface in apical area (*P*=0.051), but the difference was significant at middle and coronal areas (*P*=0.001). In middle and coronal areas it was found that dentine-sealer interface was better in BC Sealer group than AH-Plus group, and both outperformed Dorifill group.

Evaluation of Spearman’s rho (correlation coefficient) showed significant correlation between gap width and dentine-sealer interface quality (*P*<0.001). Since this coefficient was calculated as -0.418, it can be said that the lower gap width, the better the quality of dentine-sealer interface. Tubular penetration of sealers was observed only in AH-Plus group. 

## Discussion

The present *in vitro* SEM study showed that BC Sealer and AH-Plus had less gaps than Dorifill in coronal areas of the canal. In addition, BC Sealer had better dentine interface in middle and coronal area compared to AH-Plus, and both performed better than Dorifill. Application of sealers with appropriate properties such as adhesion, adaptation and tubular penetration brings two positive outcomes. First, establishment of canal sealing due to higher sealer interface with dentine wall [19]; second, entombment of residual bacteria in dentine tubules which is actually the anti-bacterial effect of sealers [20, 21]. 

Among extensive range of sealers available for root treatment, Endosequence BC Sealer has desirable properties such as osteoconductivity, being hydrophilic, having adhesion and ability to form chemical bond with the dentine walls of the root canal, *etc. *[8]. AH-Plus sealer on the other hand, is well adapted to the root canal walls, penetrates the dentinal tubules and is better than ZOE based or silicon based sealers [10]. 

In this study, EDTA was used for elimination of smear layer. It easily enters dentine tubules due to low surface tension and eliminates smear layer up to the depth of 2.5-4 µm [22]. Thus bonding and adaptation of sealer to root walls is increased [23]. Finally, distilled water was used to compensate the lasting impact of irrigations used. Findings of the current study suggested that BC Sealer and AH-Plus groups showed lower gap width compared to Dorifill group. In addition, canals filled by BC Sealer showed better dentine interface in middle and coronal areas compared to AH-Plus and both were better than Dorifill sealer, which showed results consistent to the study by Pawar *et al. *[17]. In their study, BC Sealers and Epiphany sealer had better apical seal in root canal compared to AH-Plus. Of course, the study was done by dye penetration, which is different from the current study [17]. Also in another SEM study for evaluation of gap between dentine and sealers, AH-Plus sealer showed larger gaps compared to BC Sealer and Gutta flow [18].

On the other hand, AH-Plus had the best dentine adaptation and tubular penetration using SEM compared to a ZOE-based sealer, calcium hydroxide, glass ionomer and silicone based sealers [10]. 

ZOE-based sealers have weaker bonding with root dentine compared to sealers with epoxy resin based sealers like AH-Plus, because resin sealers create mechanical retention between sealer and root dentine leading to more adhesion [24-26]. Today various sealers have replaced ZOE-based sealers with better sealing properties. Filling the canal with gutta-percha and ZOE-based sealers is considered bellow the standard of care. [27, 28]. 

**Table 1:** Descriptive statistics on the canal and sealer gaps width in the experimental groups and different areas

**Groups **		**Mean (SD)**	**Minimum**	**Maximum**
**Dorifill**	**Apical**	6.1927 (5.099)	0.00	15.65
	**Middle**	7.9999 (6.81)	0.00	19.07
	**Coronal**	12.6427 (8.62)	0.00	26.50
**AH-Plus**	**Apical**	8.0556 (7.54)	2.55	27.03
	**Middle**	5.6335 (2.56)	0.00	9.49
	**Coronal**	3.2679 (2.95)	0.00	8.42
**BC Sealer**	**Apical**	4.2950 (4.36)	0.00	10.76
	**Middle**	6.6227 (6.43)	0.00	17.64
	**Coronal**	4.4897 (8.37)	0.00	26.60

Sealing ability of a calcium phosphate-based sealers (Capseal), AH-Plus and Pulp canal sealer (ZOE-based) has been evaluated with bacterial leakage model. Leakage of three sealers was similar [29], which is in contrast to findings in the present study, because ZOE-based sealer had higher voids that can be explained by use of different methods in these two studies. In a different SEM evaluation of sealer adaptation with root canal walls, Capseal and AH-Plus sealers showed good results [29], which is consistent with current study, showing that sealer-dentine interface in resin and bioceramic sealers was better than ZOE-based sealer. 

The result of one SEM study showed that apical seal of AH-Plus and iRoot sealers (another bioceramic sealer) with single cone technique was similar [15]. However, adaptation of iRoot sealer with gutta-percha was better than AH-Plus [15] which is consistent with current study.

Sealer-dentine interface is a critical area in obturated root canals [30]. Sealers with epoxy resin [31] and bioceramic bases [15] do not shrink during setting, and it can be reason for their suitable adaptation in the gap between dentine and sealer compared to ZOE-based sealer [32].

Presence of void and gaps within the filling material can be a result of lateral condensation filling technique which does not allow creation of a homogeneous layer of sealer to the entire length of the canal [33].

In the current study, three types of sealers with three different bases were used for comparison of root canal wall dentine interface. AH-Plus sealer is widely used [34], but the comparison of this sealer with newer bioceramic sealers like BC sealer has been done rarely [17, 18]. Results of this study suggest that there is a relationship between quantitative results obtained for gap width in different groups and qualitative results obtained from dentine-sealer interface quality. Findings of this study supplement previous studies on positive characteristics of bio-ceramic sealers and emphasize on the necessity for further future studies about the characteristics of these sealers.

## Conclusion

Considering the limitations of this study, the gaps in sealer-dentine interface had lower width in BC Sealer and AH-Plus samples compared to Dorifill sealer. Interface of BC Sealer and dentine wall in middle and coronal area of root was better than AH-Plus and both were superior to Dorifill sealer. There was inverse relationship between width of the gaps and the quality of dentine–sealer interface.
